# Poly[μ-aqua-diaquabis­[μ-2-cyano-2-(oxidoimino)­acetato]­copper(II)dipotassium]

**DOI:** 10.1107/S1600536812036641

**Published:** 2012-09-26

**Authors:** Irina A. Golenya, Yulia A. Izotova, Natalia I. Usenko, Valentina A. Kalibabchuk, Natalia V. Kotova

**Affiliations:** a Kiev National Taras Shevchenko University, Department of Chemistry, Volodymyrska Str. 64, 01601 Kiev, Ukraine; b Department of Chemistry, Saint-Petersburg State University, Universitetsky Pr. 26, 198504 Stary Petergof, Russian Federation; c Department of General Chemistry, O. O. Bohomolets National Medical University, Shevchenko Blvd. 13, 01601 Kiev, Ukraine

## Abstract

In the title compound, [CuK_2_(C_3_N_2_O_3_)_2_(H_2_O)_3_]_*n*_, the Cu^2+^ atom is in a distorted square-pyramidal coordination geometry. Two N atoms belonging to the oxime groups and two O atoms belonging to the carboxyl­ate groups of two *trans*-disposed doubly deprotonated residues of 2-cyano-2-(hy­droxy­imino)­acetic acid make up the basal plane and the apical position is occupied by the water mol­ecule. The neighboring Cu-containing moieties are linked into a three-dimensional framework by K—O and K—N contacts formed by two potassium cations with the carboxyl­ate and the oxime O atoms and the nitrile N atoms of the ligand. The environments of the K^+^ cations are complemented to octa- and nona­coordinated, by K—O contacts with H_2_O mol­ecules. The crystal structure features O—H⋯O hydrogen bonds.

## Related literature

For the use of mononuclear complexes in the preparation of polynuclear complexes, see: Kahn (1993 ▶); Goodwin *et al.* (2000 ▶); Krämer & Fritsky (2000 ▶); Fritsky *et al.* (2001 ▶, 2003 ▶); Wörl *et al.* (2005 ▶). For the use of derivatives of 2-hy­droxy­imino­carb­oxy­lic acids and their derivatives as versatile ligands, see: Dvorkin *et al.* (1990*a* ▶,*b* ▶); Lampeka *et al.* (1989 ▶); Skopenko *et al.* (1990 ▶); Sachse *et al.* (2008 ▶); Fritsky *et al.* (1998 ▶, 2006 ▶); Kanderal *et al.* (2005 ▶); Moroz *et al.* (2008 ▶, 2010 ▶, 2012 ▶). For metal complexes of 2-cyano-2-(hy­droxy­imino)­acetic acid, see: Sliva *et al.* (1998 ▶); Mokhir *et al.* (2002 ▶); Eddings *et al.* (2004 ▶). For related structures, see: Duda *et al.* (1997 ▶); Fritsky *et al.* (2004 ▶); Onindo *et al.* (1995 ▶); Sliva *et al.* (1997 ▶); Świątek-Kozłowska *et al.* (2000 ▶); Kovbasyuk *et al.* (2004 ▶). For the synthesis of the ligand, see: Sliva *et al.* (1998 ▶).
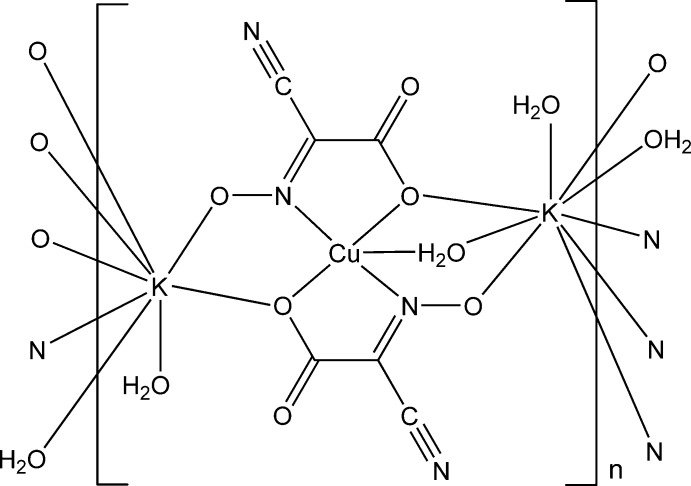


## Experimental

### 

#### Crystal data

[CuK_2_(C_3_N_2_O_3_)_2_(H_2_O)_3_]
*M* *_r_* = 419.89
Monoclinic, 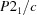

*a* = 8.767 (2) Å
*b* = 12.426 (3) Å
*c* = 13.159 (5) Å
β = 108.26 (3)°
*V* = 1361.3 (7) Å^3^
*Z* = 4
Mo *K*α radiation
μ = 2.27 mm^−1^
*T* = 100 K
0.24 × 0.16 × 0.07 mm

#### Data collection

Nonius KappaCCD area-detector diffractometer
Absorption correction: multi-scan (*DENZO*/*SCALEPACK*; Otwinowski & Minor, 1997 ▶) *T*_min_ = 0.657, *T*_max_ = 0.859
9166 measured reflections
3189 independent reflections
3006 reflections with *I* > 2σ(*I*)
*R*_int_ = 0.043

#### Refinement

*R*[*F* ^2^ > 2σ(*F* ^2^)] = 0.024
*wR*(*F* ^2^) = 0.062
*S* = 1.09
3189 reflections
205 parameters
4 restraints
H-atom parameters constrained
Δρ_max_ = 0.56 e Å^−3^
Δρ_min_ = −0.56 e Å^−3^

### 

Data collection: *COLLECT* (Nonius, 2000 ▶); cell refinement: *DENZO*/*SCALEPACK* (Otwinowski & Minor, 1997 ▶); data reduction: *DENZO*/*SCALEPACK*; program(s) used to solve structure: *SIR2004* (Burla *et al.*, 2005 ▶); program(s) used to refine structure: *SHELXL97* (Sheldrick, 2008 ▶); molecular graphics: *DIAMOND* (Brandenburg, 2009 ▶); software used to prepare material for publication: *SHELXL97*.

## Supplementary Material

Crystal structure: contains datablock(s) global, I. DOI: 10.1107/S1600536812036641/hp2047sup1.cif

Structure factors: contains datablock(s) I. DOI: 10.1107/S1600536812036641/hp2047Isup2.hkl

Additional supplementary materials:  crystallographic information; 3D view; checkCIF report

## Figures and Tables

**Table 1:** Hydrogen-bond geometry (Å, °)

*D*—H⋯*A*	*D*—H	H⋯*A*	*D*⋯*A*	*D*—H⋯*A*
O1*W*—H11*W*⋯O3*A* ^i^	0.89	1.85	2.7257 (19)	171
O1*W*—H21*W*⋯O3^ii^	0.80	1.96	2.6910 (19)	151
O2*W*—H12*W*⋯O1^iii^	0.81	2.19	2.993 (2)	173
O2*W*—H22*W*⋯O3*A* ^i^	0.92	2.02	2.926 (2)	164

Symmetry codes: (i) 
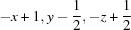
; (ii) 
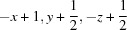
; (iii) 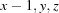.

**Fig. 1.**
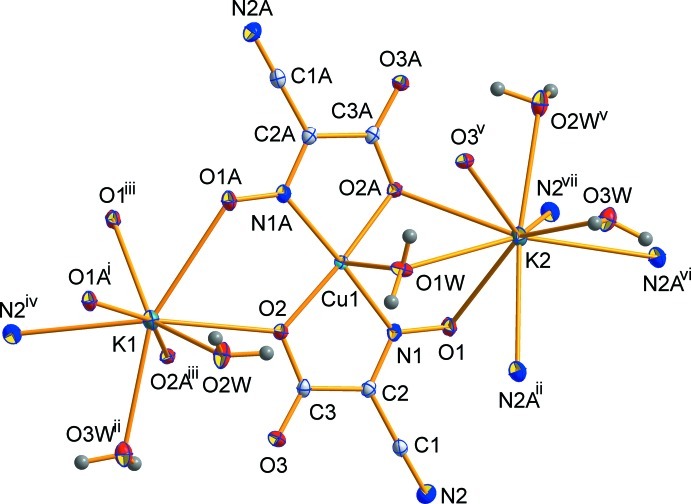
A view of compound (I), with displacement ellipsoids shown at the 50% probability level. H atoms are drawn as spheres of arbitrary radii. Symmetry codes: (i) -x, -y + 1, -z; (ii) -x + 1, y - 1/2, -z + 1/2; (iii) -x + 1, -y + 1, -z; (iv) x - 1, -y + 1/2, z - 1/2; (v) -x + 1, y + 1/2, -z + 1/2; (vi) x + 1, -y + 3/2, z + 1/2; (vii) -x + 2, y + 1/2, -z + 1/2.

**Fig. 2.**
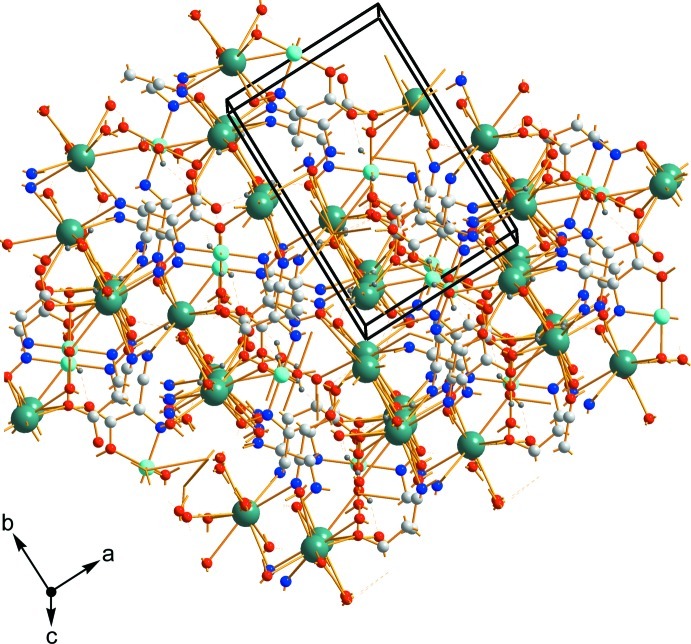
A packing diagram of the title compound. Hydrogen bonds are indicated by dashed lines.

## supplementary crystallographic information

### Comment

Many reported mononuclear complexes of 3 d-metals contains vacant donor atoms or chelate centers, so that they can be considered as ligands for preparation of homo- and heteropolynuclear systems which are widely used in bioinorganic modeling, catalysis and in molecular magnetism (Kahn, 1993; Goodwin *et al.*, 2000; Krämer *et al.*, 2000; Fritsky *et al.*, 2001; Fritsky *et al.*, 2003; Wörl *et al.*, 2005). Polydentate ligands containing oxime and carboxylic groups attract particular attention due to their potential for the bridging mode of coordination and mediation of strong magnetic exchange interactions between metal ions (Lampeka *et al.*, 1989; Dvorkin *et al.*, 1990*a*, 1990*b*; Skopenko *et al.*, 1990; Sachse *et al.*, 2008; Moroz *et al.*, 2008, 2010, 2012) and for preparation of metal complexes with efficient stabilization of unusually high oxidation states of 3 d-metal ions like copper(III) and nickel(III) (Fritsky *et al.*, 1998; Kanderal *et al.*, 2005; Fritsky *et al.*, 2006). 2-cyano-2-(hydroxyimino)acetic acid (aaco) is an efficient chelating ligand for Cu(II) and Ni(II) ions (Sliva *et al.*, 1998; Mokhir *et al.*, 2002). To date, only one heterometallic complex containing this ligand K_2_[Pd(aaco-2H)_2_].4H_2_O has been structurally characterized (Eddings *et al.*, 2004). Herein we report the second heterometallic complex based on 2-cyan-2-hydroxyiminoacetic acid.

The title compound, [K_2_Cu(C_3_N_2_O_3_)_2_(H_2_O)_3_]*_n_*, has an ionic structure containing 2- charged Cu(II)-centered complex anions, potassium cations and water molecules (Fig. 1). The Cu atom is in a distorted square-pyramidal geometry, defined by two N atoms belonging to the oxime groups and two O atoms belonging to the carboxylic groups of two *trans*-disposed doubly deprotonated residues of 2-cyano-2-(hydroxyimino)acetic acid. The apical position is occupied by the water molecule O1W which also serves as a bridge between Cu1 and K1 ions. The coordination bond lengths Cu—N and Cu—O (Table 1) are typical for square-pyramidal Cu(II) complexes with deprotonated oxime and carboxylate donors (Sliva *et al.*, 1997; Kanderal *et al.*, 2005). The bite angles around the central atom deviate from an ideal square-planar configuration [*e.g.* O2—Cu1—N1 = 82.89 (6)°], which is a consequence of the formation of five-membered chelate rings. The bond lengths C—O, N—O and C—N in the coordinated 2-oximinocarboxylate ligand are typical for copper(II) complexes with cyanoximes and carboxylates (Onindo *et al.*, 1995; Duda *et al.*, 1997; Fritsky *et al.*, 2004;).

The potassium cations K1 and K2 are bound to the copper(II) complex anion in a chelate fashion *via* the oxime oxygen (O1A and O1, respectively) and the carboxylic oxygen (O2 and O2A, respectively) atoms. Such coordination of two potassium cations from the different side of the complex anion results in a closed metallamacrocylic framework. Both potassium cations also forms additional K—O and K—N contacts with the carboxylic and the oxime O atoms and the nitrile N atoms of the neighboring Cu complex anions thus uniting them in a three-dimensional framework (Fig. 2). The environments of K1 and K2 potassium cation are complemented to octa- and nona-coordinated, respectively, by K—O contacts with H_2_O molecules. The K—O and K—N bond lengths are normal for potassium cations and close to those reported in the structures of the carboxylate and the oximate complexes (Fritsky *et al.*, 1998; Świątek-Kozłowska *et al.*, 2000; Kovbasyuk *et al.*, 20040). The crystal structure involves intermolecular O—H···O hydrogen bonds where the water molecules act as donors, and the carboxylic and the oxime O atoms act as acceptors (Table 2).

### Experimental

Cu(NO_3_)_2_.3H_2_O (0.242 g, 1 mmol) was dissolved in water (3 ml) and added to the methanolic solution (15 ml) of 2-cyano-2-(hydroxyimino)acetic acid (0.228 g, 2 mmol), synthesizsed according to Sliva *et al.*, 1998). To the obtained mixture, aqueous solution of potassium hydroxide (1*M*, 4 ml) was added with vigorous stirring at room temperature. The obtained transparent solution was stirred 20 min. and then set aside for crystallization at ambient temperature. Bright brown crystals were separated by filtration after 72 h, washed with cold water (10 ml) and dried (yield 78%). Analysis calculated for C_6_H_6_Cu *K*_2_N_4_O_9_: C 17.16, H 1.44, N 13.34%; found: C 17.10, H 1.53, N 13.42%.

### Refinement

The H atoms of the water molecule were located at the difference Fourier map and their coordinates were allowed to ride on the coordinates of the parent atom with *U*_iso_(H) = 1.5*U*_eq_.

### Crystal data

**Table:**

[CuK_2_(C_3_N_2_O_3_)_2_(H_2_O)_3_]	*F*(000) = 836
*M**_r_* = 419.89	*D*_x_ = 2.049 Mg m^−^^3^
Monoclinic, *P*2_1_/*c*	Mo *K*α radiation, λ = 0.71073 Å
Hall symbol: -P 2ybc	Cell parameters from 3744 reflections
*a* = 8.767 (2) Å	θ = 1.0–27.5°
*b* = 12.426 (3) Å	µ = 2.27 mm^−^^1^
*c* = 13.159 (5) Å	*T* = 100 K
β = 108.26 (3)°	Block, brown
*V* = 1361.3 (7) Å^3^	0.24 × 0.16 × 0.07 mm
*Z* = 4	

### Data collection

**Table:**

Nonius KappaCCD area-detector diffractometer	3189 independent reflections
Radiation source: fine-focus sealed tube	3006 reflections with *I* > 2σ(*I*)
Graphite monochromator	*R*_int_ = 0.043
ω scans	θ_max_ = 28.4°, θ_min_ = 3.7°
Absorption correction: multi-scan (*DENZO*/*SCALEPACK*; Otwinowski & Minor, 1997)	*h* = −11→11
*T*_min_ = 0.657, *T*_max_ = 0.859	*k* = −15→15
9166 measured reflections	*l* = −14→17

### Refinement

**Table:**

Refinement on *F*^2^	Primary atom site location: structure-invariant direct methods
Least-squares matrix: full	Secondary atom site location: difference Fourier map
*R*[*F*^2^ > 2σ(*F*^2^)] = 0.024	Hydrogen site location: inferred from neighbouring sites
*wR*(*F*^2^) = 0.062	H-atom parameters constrained
*S* = 1.09	*w* = 1/[σ^2^(*F*_o_^2^) + (0.028*P*)^2^ + 0.9392*P*] where *P* = (*F*_o_^2^ + 2*F*_c_^2^)/3
3189 reflections	(Δ/σ)_max_ = 0.001
205 parameters	Δρ_max_ = 0.56 e Å^−^^3^
4 restraints	Δρ_min_ = −0.56 e Å^−^^3^

### Special details

**Table:**

Geometry. All e.s.d.'s (except the e.s.d. in the dihedral angle between two l.s. planes) are estimated using the full covariance matrix. The cell e.s.d.'s are taken into account individually in the estimation of e.s.d.'s in distances, angles and torsion angles; correlations between e.s.d.'s in cell parameters are only used when they are defined by crystal symmetry. An approximate (isotropic) treatment of cell e.s.d.'s is used for estimating e.s.d.'s involving l.s. planes.
Refinement. Refinement of *F*^2^ against ALL reflections. The weighted *R*-factor *wR* and goodness of fit *S* are based on *F*^2^, conventional *R*-factors *R* are based on *F*, with *F* set to zero for negative *F*^2^. The threshold expression of *F*^2^ > σ(*F*^2^) is used only for calculating *R*-factors(gt) *etc*. and is not relevant to the choice of reflections for refinement. *R*-factors based on *F*^2^ are statistically about twice as large as those based on *F*, and *R*- factors based on ALL data will be even larger.

### Fractional atomic coordinates and isotropic or equivalent isotropic displacement parameters (Å^2^)

**Table:**

	*x*	*y*	*z*	*U*_iso_*/*U*_eq_	
Cu1	0.53009 (2)	0.529307 (15)	0.136932 (15)	0.00981 (7)	
K1	0.10262 (4)	0.38634 (3)	−0.06404 (3)	0.01425 (9)	
K2	0.86027 (5)	0.61378 (3)	0.38164 (3)	0.01645 (9)	
O1	0.86577 (14)	0.45133 (10)	0.22512 (10)	0.0138 (2)	
O1A	0.20044 (14)	0.60207 (10)	0.03346 (10)	0.0151 (2)	
O2	0.41091 (14)	0.39928 (10)	0.08061 (10)	0.0139 (2)	
O2A	0.65548 (14)	0.66325 (10)	0.16771 (9)	0.0117 (2)	
O3	0.43256 (15)	0.22059 (10)	0.09720 (10)	0.0160 (3)	
O3A	0.61938 (14)	0.84124 (10)	0.16679 (10)	0.0140 (2)	
O1W	0.52488 (16)	0.52800 (10)	0.30436 (10)	0.0160 (3)	
H11W	0.4781 (7)	0.4699 (8)	0.3201 (2)	0.024*	
H21W	0.5029 (3)	0.5835 (8)	0.3278 (3)	0.024*	
O2W	0.10231 (16)	0.33982 (12)	0.13939 (11)	0.0205 (3)	
H12W	0.0333 (11)	0.3648 (4)	0.1612 (4)	0.031*	
H22W	0.1900 (14)	0.3541 (3)	0.1987 (10)	0.031*	
O3W	0.89136 (15)	0.65821 (11)	0.59001 (11)	0.0194 (3)	
H13W	0.8132 (13)	0.6471 (2)	0.6103 (4)	0.029*	
H23W	0.9754 (14)	0.6349 (4)	0.6428 (9)	0.029*	
N1	0.71631 (17)	0.42739 (12)	0.18069 (11)	0.0111 (3)	
N1A	0.34617 (17)	0.62774 (12)	0.07855 (11)	0.0113 (3)	
N2	0.86325 (19)	0.16875 (13)	0.23004 (13)	0.0203 (3)	
N2A	0.19141 (19)	0.88673 (13)	0.03683 (13)	0.0186 (3)	
C1	0.7741 (2)	0.23825 (14)	0.20181 (13)	0.0125 (3)	
C1A	0.2811 (2)	0.81670 (14)	0.06281 (13)	0.0128 (3)	
C2	0.66627 (19)	0.32726 (14)	0.16742 (13)	0.0111 (3)	
C2A	0.3926 (2)	0.72992 (14)	0.09515 (13)	0.0114 (3)	
C3	0.4904 (2)	0.31178 (13)	0.11168 (13)	0.0114 (3)	
C3A	0.5671 (2)	0.74813 (14)	0.14657 (13)	0.0109 (3)	

### Atomic displacement parameters (Å^2^)

**Table:**

	*U*^11^	*U*^22^	*U*^33^	*U*^12^	*U*^13^	*U*^23^
Cu1	0.00914 (11)	0.00802 (11)	0.01111 (11)	0.00006 (7)	0.00152 (8)	−0.00062 (7)
K1	0.01241 (17)	0.01672 (18)	0.01307 (17)	−0.00156 (13)	0.00320 (13)	−0.00110 (13)
K2	0.01638 (18)	0.01814 (19)	0.01222 (17)	0.00000 (14)	0.00070 (14)	−0.00195 (13)
O1	0.0092 (5)	0.0152 (6)	0.0157 (6)	−0.0015 (4)	0.0020 (5)	−0.0016 (5)
O1A	0.0103 (6)	0.0166 (6)	0.0168 (6)	−0.0026 (5)	0.0019 (5)	−0.0013 (5)
O2	0.0106 (5)	0.0111 (6)	0.0173 (6)	0.0003 (4)	0.0006 (5)	−0.0008 (4)
O2A	0.0103 (5)	0.0101 (5)	0.0142 (6)	0.0007 (4)	0.0029 (4)	0.0002 (4)
O3	0.0155 (6)	0.0104 (6)	0.0197 (6)	−0.0020 (5)	0.0021 (5)	−0.0001 (5)
O3A	0.0138 (6)	0.0112 (6)	0.0165 (6)	−0.0003 (5)	0.0037 (5)	−0.0016 (5)
O1W	0.0230 (7)	0.0095 (6)	0.0175 (6)	−0.0016 (5)	0.0093 (5)	−0.0011 (4)
O2W	0.0131 (6)	0.0311 (7)	0.0163 (6)	0.0019 (5)	0.0033 (5)	−0.0007 (5)
O3W	0.0145 (6)	0.0269 (7)	0.0169 (6)	0.0023 (5)	0.0049 (5)	0.0017 (5)
N1	0.0110 (6)	0.0134 (7)	0.0088 (6)	0.0010 (5)	0.0030 (5)	−0.0006 (5)
N1A	0.0103 (6)	0.0138 (7)	0.0098 (6)	−0.0007 (5)	0.0034 (5)	−0.0002 (5)
N2	0.0195 (8)	0.0178 (8)	0.0200 (8)	0.0028 (6)	0.0010 (6)	0.0000 (6)
N2A	0.0147 (7)	0.0180 (8)	0.0212 (8)	0.0026 (6)	0.0029 (6)	0.0006 (6)
C1	0.0118 (7)	0.0140 (8)	0.0103 (7)	−0.0018 (6)	0.0015 (6)	−0.0009 (6)
C1A	0.0120 (7)	0.0149 (8)	0.0110 (7)	−0.0017 (6)	0.0028 (6)	−0.0017 (6)
C2	0.0115 (7)	0.0123 (7)	0.0093 (7)	0.0009 (6)	0.0030 (6)	0.0000 (6)
C2A	0.0117 (8)	0.0131 (8)	0.0094 (7)	0.0021 (6)	0.0036 (6)	0.0000 (6)
C3	0.0111 (7)	0.0140 (8)	0.0085 (7)	−0.0004 (6)	0.0023 (6)	0.0006 (6)
C3A	0.0112 (7)	0.0139 (8)	0.0080 (7)	0.0014 (6)	0.0038 (6)	0.0008 (6)

### Geometric parameters (Å, º)

**Table:**

Cu1—O2	1.9407 (14)	O2—C3	1.287 (2)
Cu1—O2A	1.9656 (14)	O2A—C3A	1.287 (2)
Cu1—N1A	1.9774 (16)	O2A—K1^iii^	2.9244 (15)
Cu1—N1	2.0029 (16)	O3—C3	1.231 (2)
Cu1—O1W	2.2181 (15)	O3—K2^ii^	2.9795 (16)
K1—O2W	2.7395 (17)	O3A—C3A	1.242 (2)
K1—O2	2.7803 (16)	O1W—H11W	0.8863
K1—O1A^i^	2.8128 (14)	O1W—H21W	0.8027
K1—O3W^ii^	2.8578 (16)	O2W—K2^ii^	2.8513 (17)
K1—O2A^iii^	2.9244 (15)	O2W—H12W	0.8088
K1—N2^iv^	2.941 (2)	O2W—H22W	0.9250
K1—O1A	2.9795 (16)	O3W—K1^v^	2.8578 (16)
K1—O1^iii^	3.0011 (16)	O3W—H13W	0.8215
K2—O3W	2.7255 (17)	O3W—H23W	0.8882
K2—O2W^v^	2.8513 (17)	N1—C2	1.313 (2)
K2—O2A	2.8911 (18)	N1—K1^iii^	3.4294 (18)
K2—O1	2.8951 (16)	N1A—C2A	1.330 (2)
K2—O3^v^	2.9795 (16)	N2—C1	1.146 (2)
K2—N2A^vi^	2.981 (2)	N2—K1^viii^	2.941 (2)
K2—O1W	2.9904 (17)	N2—K2^ix^	3.2763 (19)
K2—N2A^ii^	3.1018 (19)	N2A—C1A	1.151 (2)
K2—N2^vii^	3.2763 (19)	N2A—K2^x^	2.981 (2)
K2—N1	3.444 (2)	N2A—K2^v^	3.1018 (18)
O1—N1	1.2911 (19)	C1—C2	1.433 (2)
O1—K1^iii^	3.0011 (16)	C1A—C2A	1.428 (2)
O1A—N1A	1.2692 (19)	C2—C3	1.498 (2)
O1A—K1^i^	2.8128 (14)	C2A—C3A	1.483 (2)
			
O2—Cu1—O2A	169.44 (5)	O2W^v^—K2—N2^vii^	68.12 (5)
O2—Cu1—N1A	95.20 (6)	O2A—K2—N2^vii^	80.79 (5)
O2A—Cu1—N1A	83.78 (6)	O1—K2—N2^vii^	69.31 (5)
O2—Cu1—N1	82.89 (6)	O3^v^—K2—N2^vii^	136.83 (4)
O2A—Cu1—N1	97.08 (6)	N2A^vi^—K2—N2^vii^	66.99 (5)
N1A—Cu1—N1	174.16 (6)	O1W—K2—N2^vii^	135.12 (4)
O2—Cu1—O1W	101.37 (6)	N2A^ii^—K2—N2^vii^	123.61 (5)
O2A—Cu1—O1W	89.19 (6)	O3W—K2—Cu1	136.80 (4)
N1A—Cu1—O1W	97.03 (6)	O2W^v^—K2—Cu1	105.92 (4)
N1—Cu1—O1W	88.76 (6)	O2A—K2—Cu1	31.24 (3)
O2—Cu1—K2	137.80 (4)	O1—K2—Cu1	51.18 (3)
O2A—Cu1—K2	49.71 (4)	O3^v^—K2—Cu1	75.44 (4)
N1A—Cu1—K2	118.52 (5)	N2A^vi^—K2—Cu1	156.01 (4)
N1—Cu1—K2	65.73 (5)	O1W—K2—Cu1	36.34 (3)
O1W—Cu1—K2	53.03 (4)	N2A^ii^—K2—Cu1	83.40 (5)
O2—Cu1—K1^iii^	122.19 (4)	N2^vii^—K2—Cu1	98.86 (4)
O2A—Cu1—K1^iii^	49.64 (4)	N1—K2—Cu1	32.02 (3)
N1A—Cu1—K1^iii^	112.66 (5)	O3W—K2—K1^v^	43.95 (4)
N1—Cu1—K1^iii^	64.30 (5)	O2W^v^—K2—K1^v^	41.71 (3)
O1W—Cu1—K1^iii^	122.53 (4)	O2A—K2—K1^v^	107.77 (4)
K2—Cu1—K1^iii^	69.53 (3)	O1—K2—K1^v^	167.26 (3)
O2W—K1—O2	69.00 (5)	O3^v^—K2—K1^v^	59.37 (4)
O2W—K1—O1A^i^	65.22 (5)	N2A^vi^—K2—K1^v^	73.70 (5)
O2—K1—O1A^i^	131.17 (4)	O1W—K2—K1^v^	112.48 (4)
O2W—K1—O3W^ii^	85.02 (5)	N2A^ii^—K2—K1^v^	122.80 (4)
O2—K1—O3W^ii^	95.09 (5)	N2^vii^—K2—K1^v^	98.98 (4)
O1A^i^—K1—O3W^ii^	96.98 (5)	N1—K2—K1^v^	161.23 (3)
O2W—K1—O2A^iii^	129.37 (5)	Cu1—K2—K1^v^	129.25 (3)
O2—K1—O2A^iii^	68.90 (4)	O3W—K2—H21W	91.8
O1A^i^—K1—O2A^iii^	158.93 (4)	O2W^v^—K2—H21W	104.1
O3W^ii^—K1—O2A^iii^	72.05 (4)	O2A—K2—H21W	61.0
O2W—K1—N2^iv^	129.21 (5)	O1—K2—H21W	89.6
O2—K1—N2^iv^	154.70 (5)	O3^v^—K2—H21W	38.2
O1A^i^—K1—N2^iv^	73.17 (5)	N2A^vi^—K2—H21W	151.5
O3W^ii^—K1—N2^iv^	72.16 (5)	O1W—K2—H21W	15.4
O2A^iii^—K1—N2^iv^	86.19 (5)	N2A^ii^—K2—H21W	73.4
O2W—K1—O1A	81.80 (5)	N2^vii^—K2—H21W	141.4
O2—K1—O1A	64.30 (5)	N1—K2—H21W	68.4
O1A^i^—K1—O1A	92.85 (4)	Cu1—K2—H21W	45.2
O3W^ii^—K1—O1A	158.48 (4)	K1^v^—K2—H21W	97.3
O2A^iii^—K1—O1A	103.74 (4)	N1—O1—K2	104.00 (9)
N2^iv^—K1—O1A	129.20 (5)	N1—O1—K1^iii^	98.06 (9)
O2W—K1—O1^iii^	149.39 (4)	K2—O1—K1^iii^	93.42 (5)
O2—K1—O1^iii^	99.18 (5)	N1A—O1A—K1^i^	141.06 (10)
O1A^i^—K1—O1^iii^	111.53 (5)	N1A—O1A—K1	122.63 (10)
O3W^ii^—K1—O1^iii^	124.96 (4)	K1^i^—O1A—K1	87.15 (4)
O2A^iii^—K1—O1^iii^	64.61 (4)	C3—O2—Cu1	114.14 (11)
N2^iv^—K1—O1^iii^	72.71 (5)	C3—O2—K1	118.81 (10)
O1A—K1—O1^iii^	67.77 (4)	Cu1—O2—K1	126.95 (6)
O2W—K1—Cu1^iii^	124.58 (4)	C3A—O2A—Cu1	112.93 (11)
O2—K1—Cu1^iii^	55.64 (4)	C3A—O2A—K2	122.22 (10)
O1A^i^—K1—Cu1^iii^	159.87 (3)	Cu1—O2A—K2	99.04 (6)
O3W^ii^—K1—Cu1^iii^	101.26 (4)	C3A—O2A—K1^iii^	123.20 (10)
O2A^iii^—K1—Cu1^iii^	30.81 (3)	Cu1—O2A—K1^iii^	99.55 (5)
N2^iv^—K1—Cu1^iii^	104.42 (4)	K2—O2A—K1^iii^	95.14 (5)
O1A—K1—Cu1^iii^	72.96 (4)	C3—O3—K2^ii^	136.08 (11)
O1^iii^—K1—Cu1^iii^	50.26 (3)	Cu1—O1W—K2	90.63 (5)
N1^iii^—K1—Cu1^iii^	31.75 (3)	Cu1—O1W—H11W	113.2
O2W—K1—K1^i^	66.33 (4)	K2—O1W—H11W	133.5
O2—K1—K1^i^	98.05 (4)	Cu1—O1W—H21W	117.2
O1A^i^—K1—K1^i^	48.16 (3)	K2—O1W—H21W	83.8
O3W^ii^—K1—K1^i^	141.22 (3)	H11W—O1W—H21W	115.1
O2A^iii^—K1—K1^i^	146.54 (3)	K1—O2W—K2^ii^	94.45 (5)
N2^iv^—K1—K1^i^	105.52 (5)	K1—O2W—H12W	119.7
O1A—K1—K1^i^	44.69 (3)	K2^ii^—O2W—H12W	122.3
O1^iii^—K1—K1^i^	88.64 (4)	K1—O2W—H22W	122.0
N1^iii^—K1—K1^i^	92.55 (3)	K2^ii^—O2W—H22W	100.4
Cu1^iii^—K1—K1^i^	116.27 (3)	H12W—O2W—H22W	98.2
O2W—K1—K2^ii^	43.83 (4)	K2—O3W—K1^v^	94.61 (5)
O2—K1—K2^ii^	76.44 (4)	K2—O3W—H13W	117.5
O1A^i^—K1—K2^ii^	82.18 (4)	K1^v^—O3W—H13W	104.7
O3W^ii^—K1—K2^ii^	41.44 (3)	K2—O3W—H23W	121.2
O2A^iii^—K1—K2^ii^	99.36 (4)	K1^v^—O3W—H23W	112.0
N2^iv^—K1—K2^ii^	104.45 (5)	H13W—O3W—H23W	105.3
O1A—K1—K2^ii^	122.09 (4)	O1—N1—C2	121.88 (14)
O1^iii^—K1—K2^ii^	163.67 (3)	O1—N1—Cu1	127.20 (11)
N1^iii^—K1—K2^ii^	148.83 (3)	C2—N1—Cu1	110.65 (11)
Cu1^iii^—K1—K2^ii^	117.31 (3)	O1—N1—K1^iii^	60.05 (8)
K1^i^—K1—K2^ii^	107.48 (3)	C2—N1—K1^iii^	139.68 (11)
O3W—K2—O2W^v^	85.40 (5)	Cu1—N1—K1^iii^	83.94 (5)
O3W—K2—O2A	140.56 (4)	O1—N1—K2	54.66 (8)
O2W^v^—K2—O2A	75.60 (5)	C2—N1—K2	140.04 (11)
O3W—K2—O1	146.95 (4)	Cu1—N1—K2	82.25 (5)
O2W^v^—K2—O1	126.14 (4)	K1^iii^—N1—K2	77.30 (4)
O2A—K2—O1	66.37 (5)	O1A—N1A—C2A	121.87 (15)
O3W—K2—O3^v^	68.49 (5)	O1A—N1A—Cu1	127.23 (12)
O2W^v^—K2—O3^v^	72.48 (4)	C2A—N1A—Cu1	110.87 (11)
O2A—K2—O3^v^	72.90 (5)	C1—N2—K1^viii^	133.74 (14)
O1—K2—O3^v^	125.73 (4)	C1—N2—K2^ix^	122.50 (13)
O3W—K2—N2A^vi^	62.76 (5)	K1^viii^—N2—K2^ix^	87.14 (5)
O2W^v^—K2—N2A^vi^	87.22 (5)	C1A—N2A—K2^x^	129.07 (13)
O2A—K2—N2A^vi^	147.35 (5)	C1A—N2A—K2^v^	138.19 (13)
O1—K2—N2A^vi^	104.84 (5)	K2^x^—N2A—K2^v^	91.29 (6)
O3^v^—K2—N2A^vi^	128.30 (5)	N2—C1—C2	178.38 (19)
O3W—K2—O1W	101.07 (5)	N2A—C1A—C2A	179.9 (3)
O2W^v^—K2—O1W	116.68 (5)	N1—C2—C1	121.99 (15)
O2A—K2—O1W	60.02 (4)	N1—C2—C3	115.90 (15)
O1—K2—O1W	75.13 (5)	C1—C2—C3	122.10 (15)
O3^v^—K2—O1W	53.59 (4)	N1A—C2A—C1A	121.76 (15)
N2A^vi^—K2—O1W	151.06 (4)	N1A—C2A—C3A	116.05 (15)
O3W—K2—N2A^ii^	79.38 (5)	C1A—C2A—C3A	122.17 (15)
O2W^v^—K2—N2A^ii^	164.43 (4)	O3—C3—O2	124.94 (15)
O2A—K2—N2A^ii^	114.63 (5)	O3—C3—C2	120.30 (15)
O1—K2—N2A^ii^	69.43 (5)	O2—C3—C2	114.75 (15)
O3^v^—K2—N2A^ii^	98.59 (5)	O3A—C3A—O2A	124.08 (15)
N2A^vi^—K2—N2A^ii^	88.71 (6)	O3A—C3A—C2A	119.89 (15)
O1W—K2—N2A^ii^	63.82 (5)	O2A—C3A—C2A	116.03 (15)
O3W—K2—N2^vii^	123.65 (5)		
			
O2—Cu1—O2A—C3A	90.4 (3)	O1—N1—C2—C3	−178.21 (13)
N1A—Cu1—O2A—C3A	5.42 (11)	Cu1—N1—C2—C3	7.32 (17)
N1—Cu1—O2A—C3A	179.61 (11)	O1A—N1A—C2A—C1A	−0.2 (2)
O1W—Cu1—O2A—C3A	−91.74 (11)	Cu1—N1A—C2A—C1A	−178.39 (12)
O2—Cu1—N1—O1	175.68 (13)	O1A—N1A—C2A—C3A	−178.72 (14)
O2A—Cu1—N1—O1	6.32 (14)	Cu1—O2—C3—O3	170.18 (14)
O1W—Cu1—N1—O1	−82.70 (13)	Cu1—O2—C3—C2	−10.95 (18)
O2—Cu1—N1—C2	−10.22 (11)	N1—C2—C3—O3	−178.94 (15)
O2A—Cu1—N1—C2	−179.58 (11)	C1—C2—C3—O3	2.5 (2)
O1W—Cu1—N1—C2	91.39 (12)	N1—C2—C3—O2	2.1 (2)
O2—Cu1—N1A—O1A	7.99 (14)	C1—C2—C3—O2	−176.39 (15)
O2A—Cu1—N1A—O1A	177.43 (14)	Cu1—O2A—C3A—O3A	174.67 (13)
O1W—Cu1—N1A—O1A	−94.17 (14)	Cu1—O2A—C3A—C2A	−5.14 (17)
O2—Cu1—N1A—C2A	−173.99 (11)	N1A—C2A—C3A—O3A	−178.48 (14)
O2A—Cu1—N1A—C2A	−4.55 (11)	C1A—C2A—C3A—O3A	3.0 (2)
O1W—Cu1—N1A—C2A	83.86 (12)	N1A—C2A—C3A—O2A	1.3 (2)
O1—N1—C2—C1	0.3 (2)	C1A—C2A—C3A—O2A	−177.14 (14)
Cu1—N1—C2—C1	−174.16 (12)		

Symmetry codes: (i) −*x*, −*y*+1, −*z*; (ii) −*x*+1, *y*−1/2, −*z*+1/2; (iii) −*x*+1, −*y*+1, −*z*; (iv) *x*−1, −*y*+1/2, *z*−1/2; (v) −*x*+1, *y*+1/2, −*z*+1/2; (vi) *x*+1, −*y*+3/2, *z*+1/2; (vii) −*x*+2, *y*+1/2, −*z*+1/2; (viii) *x*+1, −*y*+1/2, *z*+1/2; (ix) −*x*+2, *y*−1/2, −*z*+1/2; (x) *x*−1, −*y*+3/2, *z*−1/2.

### Hydrogen-bond geometry (Å, º)

**Table:**

*D*—H···*A*	*D*—H	H···*A*	*D*···*A*	*D*—H···*A*
O1*W*—H11*W*···O3*A*^ii^	0.89	1.85	2.7257 (19)	171
O1*W*—H21*W*···O3^v^	0.80	1.96	2.6910 (19)	151
O2*W*—H12*W*···O1^xi^	0.81	2.19	2.993 (2)	173
O2*W*—H22*W*···O3*A*^ii^	0.92	2.02	2.926 (2)	164

Symmetry codes: (ii) −*x*+1, *y*−1/2, −*z*+1/2; (v) −*x*+1, *y*+1/2, −*z*+1/2; (xi) *x*−1, *y*, *z*.
